# Mutation analysis by deep sequencing of pancreatic juice from patients with pancreatic ductal adenocarcinoma

**DOI:** 10.1186/s12885-018-5195-7

**Published:** 2019-01-05

**Authors:** Man Hung Choi, Eline Mejlænder-Andersen, Sophia Manueldas, Khadija El Jellas, Solrun J. Steine, Kjersti Tjensvoll, Hege Aase Sætran, Stian Knappskog, Dag Hoem, Oddmund Nordgård, Randi Hovland, Anders Molven

**Affiliations:** 1 Gade Laboratory for Pathology, Department of Clinical Medicine, University of Bergen, Bergen, Norway; 2 Department of Pathology, Haukeland University Hospital, Bergen, Norway; 3 Department of Hematology and Oncology, Stavanger University Hospital, Stavanger, Norway; 4 Section of Oncology, Department of Clinical Science, University of Bergen, Bergen, Norway; 5 Department of Oncology, Haukeland University Hospital, Bergen, Norway; 6 Department of Gastrointestinal Surgery, Haukeland University Hospital, Bergen, Norway; 7 Department of Medical Genetics, Haukeland University Hospital, Bergen, Norway; 8 KG Jebsen Center for Diabetes Research, Department of Clinical Science, University of Bergen, Bergen, Norway

**Keywords:** Pancreatic cancer, *KRAS*, *TP53*, Pancreatic juice, Mutation analysis, Liquid biopsy

## Abstract

**Background:**

Reliable methods are needed to identify patients with early-stage cancer or high-grade precancerous lesions in the pancreas. Analysis of pancreatic juice to detect somatic mutations could represent one such approach. Here we investigated the concordance between mutations found in the primary tumor and pancreatic juice from the same patient.

**Methods:**

Amplicon-based targeted deep sequencing was performed on samples from 21 patients with pancreatic ductal adenocarcinoma (PDAC) who had undergone Whipple’s operation. Mutation profiles were determined in formalin-fixed sections of the primary tumor and in pancreatic juice sampled from the main pancreatic duct during surgery.

**Results:**

Using a cut-off of 3% for variant allele frequency, *KRAS* mutations were detected in 20/21 primary tumors (95%) and in 15/21 (71%) juice samples. When also considering low-frequency variants, *KRAS* mutations were found in 20/21 juice samples. Most juice samples exhibited multiple *KRAS* variants not seen in the primary tumor, and only in 11 cases (52%) did the most abundant variant of the juice correspond to the *KRAS* mutation detected in the tumor. *TP53* mutations were found in 16 tumors (76%) and six juice samples (29%). Among the positive juice samples, only one exhibited more than a single *TP53* mutation. Detection of both *KRAS* and *TP53* mutations was fully concordant in the primary tumor and juice sample in 7/21 cases (33%).

**Conclusions:**

Pancreatic juice from PDAC patients is rich in *KRAS* mutations often not seen in the primary tumor and possibly reflecting precancerous lesions in other regions of the pancreas. The inclusion of *TP53* mutation detection and additional markers must therefore be considered for fully exploiting the clinical potential of pancreatic juice samples in early cancer detection.

**Electronic supplementary material:**

The online version of this article (10.1186/s12885-018-5195-7) contains supplementary material, which is available to authorized users.

## Background

Despite many recent advances in treatment of malignant disease, pancreatic cancer remains the most lethal common solid tumor, with an overall 5-year survival rate of less than 10% [1]. The predominant histologic form of pancreatic cancer, ductal adenocarcinoma (PDAC), is biologically aggressive and often develops asymptomatically in the early course of the disease [2]. Surgical resection is the only curative option available today. However, current imaging technology is suboptimal for identifying early-stage tumors or high-grade precancerous lesions, and no clinically reliable biomarker test is available for early disease detection [3]. As a result, most patients diagnosed with PDAC present with a non-resectable advanced-stage disease and are left with only palliative treatment options. Thus, there is a strong need for progress in early detection and therapeutic approaches to improve patient outcomes in pancreatic cancer.

Deep sequencing (also known as next-generation sequencing or NGS) of circulating tumor DNA (ctDNA) in body fluids has emerged as a potential tool for cancer diagnostics and management [4]. Detection of molecular alterations in ctDNA isolated from pancreatic juice may represent a useful clinical test in pancreatic cancer diagnostics [5] as this fluid flows through the ductal system where most precursor lesions of malignant pancreatic tumors arise [3]. Early disease detection based on ctDNA should also take into account that the somatic mutations of PDAC are likely to arise in a certain temporal order because these tumors are considered to develop from defined precursor lesions, the most common being pancreatic intraepithelial neoplasia (PanIN) [6].

Exome sequencing reveals that many somatic mutations required for PDAC development, most frequently *KRAS* and *TP53*, are shared among moderate and high-grade PanINs and adjacent PDAC [7]. Oncogenic *KRAS* mutations are present in at least 90% of PDAC tumors [8], and they are likely to arise from early mutational events that occur in the large majority of low-grade PanINs (PanIN-1) [6]. Similarly, around 70% of PDAC cases harbor inactivating *TP53* mutations that arise in high-grade PanINs (PanIN-3) before they progress to invasive adenocarcinoma [6, 8]. If these and other mutations commonly present in pancreatic cancer or high-grade dysplasia could be reliably detected in pancreatic juice, there might be a potential to identify individuals with early-stage pancreatic cancer or carcinoma in situ before these lesions become visible by imaging. This may provide a window for early medical intervention and a better chance for survival.

In most reports on mutation analysis in pancreatic juice, either none or only a small number of matched tissue specimens were analyzed in parallel [9–14]. One study from 2008 reported similar mutation profiles between surgically collected pancreatic duct juice and tumor tissues from PDAC patients, but only three hotspot *KRAS* mutations were analyzed [9]. Information about concordance between tumor and juice samples with regard to *TP53* and other mutations associated with PDAC is generally scarce. Thus, it remains to be firmly established to which degree the mutations found in pancreatic juice reflect those present in the primary tumor.

In this study, our aim was to provide a better understanding of the clinical potential and challenges in early malignant disease detection by deep-sequencing-based mutational analysis of DNA isolated from pancreatic juice. We evaluated the concordance between *KRAS* and *TP53* mutation profiles in PDAC tissue and pancreatic juice sampled from the distal dilated duct during resection of the primary tumor. We found that pancreatic juice DNA harbors a panorama of *KRAS* mutations, making any diagnostic evaluation based only on this gene of limited value.

## Methods

### Collection of human pancreatic cancer specimens

We analyzed formalin-fixed, paraffin-embedded (FFPE) pancreatic tissue and pancreatic juice samples collected from 21 patients diagnosed with PDAC (Table 1). All cases (48% males, mean age: 68 years) had undergone resection of a pancreatic head tumor by the Whipple procedure at Haukeland University Hospital, Bergen, Norway between 2006 and 2016. After transection of the pancreas, the juice sample was collected by cannulating the distal, dilated duct. The sample was immediately aliquoted and stored at − 80 °C until use. For confirmation of the PDAC diagnosis, routine pathology reports were reviewed and tumor sections re-examined by a pathologist experienced in gastroenterological diseases. The study was approved by the Research Ethics Committee of Western Norway and written consent was obtained from the patients.

**Table 1:** Clinical characteristics of the 21 study patients

Case number	Age range at diagnosis (years)	Tumor size^a^ (cm)	Estimated tumor cellularity (%)	Survival (months)
1	80–89	3.5	40	10
2	60–69	2.5	45	122^b^
3	70–79	4.0	45	11
4	50–59	2.5	45	46
5	60–69	3.5	50	26
6	70–79	3.0	35	15
7	70–79	1.5	40	45
8	60–69	3.5	20	9
9	60–69	3.5	50	18
10	60–69	2.5	35	80^b^
11	50–59	2.0	50	36
12	80–89	2.5	15	19
13	70–79	3.0	50	11
14	70–79	4.0	40	11
15	70–79	4.5	50	23
16	50–59	5.0	70	10^b^
17	60–69	4.0	60	10
18	60–69	4.0	40	13
19	70–79	5.0	40	35^b^
20	60–69	2.0	30	29^b^
21	70–79	4.0	40	27

All patients had a diagnosis of pancreatic ductal carcinoma with the tumor located in the pancreatic head

^a^Largest measured dimension

^b^Patient still alive or lost to follow-up

### DNA isolation and quantification

Routine hematoxylin and eosin (H&E)-stained sections from FFPE pancreatic tumor samples were assessed for tumor cellularity by a pathologist. Areas enriched for tumor cells were identified, followed by scraping off these areas from three unstained, parallel 10-μm sections. As quality control, a final parallel 5-μm section was made from the tissue block, H&E-stained and compared with the original H&E section on which the diagnosis was based.

Tumor DNA was extracted using the QIAamp DNA FFPE Tissue kit (Qiagen) according to the manufacturer’s instructions with the following modifications to obtain higher DNA yield: Lysis of tissue was performed with 40 μl proteinase K solution per sample with overnight incubation at 56 °C. An additional volume of 30 μl proteinase K was then added and the sample further incubated at 56 °C for 2–4 h. DNA from pancreatic juice was extracted using the QIAamp DNA Investigator kit (Qiagen) as described by the manufacturer. DNA samples extracted from both specimen types were eluted in Buffer ATE provided in the kits and stored at − 20 °C until use. DNA concentration was determined on the Qubit V 3.0 fluorometer using the Qubit dsDNA BR Assay kit (Thermo Fisher Scientific).

### PCR amplification and sanger sequencing

Sequences of primers used for PCR amplification of *KRAS* exons 2 and 3, *TP53* exons 5–10 and *BRAF* exon 15 are listed in (Additional file 1: Table S1). Identical primers were used for subsequent Sanger sequencing unless otherwise specified. In general, PCR reactions were run in a total volume of 25 μl with 0.3 μM of each primer and 2 μl purified DNA using the Multiplex PCR mix (Qiagen). Q-solution from the kit was added to all reactions except for *KRAS* exon 2. The following PCR program was generally used for amplification: 95 °C for 15 min; 38 cycles of 94 °C for 1 min, T_m_ for 90 s and 72 °C for 90 s; ending with 72 °C for 10 min. T_m_ is the annealing temperature listed in (Additional file 1: Table S1). For amplification of *TP53* exons 8 and 10, touch-down PCR was performed for the first 20 three-step cycles with the annealing step decreasing from 60 °C at 0.2 °C/cycle until T_m_ was reached and then maintained for another 20 three-step cycles. PCR products were cleaned up enzymatically using the Illustra ExoProStar 1-step reagent (GE Healthcare) and sequenced in both directions. A sequencing mix of 10 μl in total with 0.2 μM primer was set up using the BigDye Terminator Cycle Sequencing kit, Version 1.1 (Applied Biosystems). The following incubation program was used: 96 °C for 1 min; 25 cycles of 96 °C for 6 s, 57 °C for 3 s and 60 °C for 4 min. Reactions were cleaned up with the BigDye XTerminator Purification kit and analyzed on the 3500xL Genetic Analyzer (both Applied Biosystems).

### Deep sequencing and data analysis

Amplicon-based targeted sequencing libraries were generated from 5 to 20 ng DNA using the TruSight Tumor 15 kit (Illumina) according to the manufacturer’s guide. This kit contains two separate primer pools to amplify, by multiplex-PCR, the hotspot or coding regions of *KRAS*, *TP53* and 13 other genes frequently mutated in solid tumors (Additional file 2: Table S2). Barcoded libraries were purified using magnetic beads provided in the kit. Each library was quantified using the Qubit dsDNA assay and checked for quality by agarose gel electrophoresis. Samples were pooled and paired-end sequenced on an Illumina MiSeq or MiniSeq sequencer, with the PhiX control (Illumina) included in each run. Bioinformatic analysis of the sequencing reads, including alignment to the hg19/GRCh37 human reference sequence and variant calling, was performed using the TruSight Tumor 15 pipeline as described in the TruSight Tumor 15 v1.0 Base Space App Guide [15]. Variants were filtered out by the pipeline before further evaluation when 1) the variant allele frequency (VAF) was < 3.0%; 2) the read depth at the variant position was <500x; 3) the quality score of the variant was < 30; 4) there was a significant strand bias, or 5) there was an indel occurring within a homopolymer region.

Variants were annotated using the software VariantStudio (Illumina). Synonymous variants were not investigated further, and neither were variants reported with an allele frequency ≥ 1% in the European or general population based on reference databases including the 1000 Genomes Project, Exome Aggregation Consortium (ExAC) and Genome Aggregation Database (gnomAD). InterVar [16] was used to aid interpretation of potential pathogenicity of variants with reference to the COSMIC and IARC *TP53* cancer mutation databases, and to prediction tools such as SIFT [17] and PolyPhen [18]. Variants were classified in accordance with the American College of Medical Genetics and Genomics guidelines [19]. Variants classified as pathogenic (class 5), likely pathogenic (class 4), and of uncertain significance (class 3) were reported if listed in COSMIC. A detailed interpretation is given in (Additional file 3: Table S3). For each identified variant, a percentage VAF was given to denote the variant allele prevalence among the total number of reads at the variant position. All reported variants were visually examined using the Integrative Genomics Viewer (IGV 2.4) [20]. Across all samples, *KRAS* codons 12, 13 and 61 were manually evaluated using the IGV for potential low-abundance variants (0.2% ≤ VAF < 3.0%). We also manually examined the *TP53* loci in the tumor-juice specimen pairs when a mutation was detected bioinformatically in either one of the samples. The low-frequency variants are specified in red text in Tables 2, 3 and 4.

### PNA clamp real-time PCR assay

Peptide nucleic acid (PNA) clamp real-time PCR was performed for independent detection of *KRAS* exon 2 mutations in DNA from pancreatic juice (5 μl) as previously described [21]. This method allows detection of *KRAS* codon 12/13 mutations with a sensitivity reaching 1 mutated allele per 10^4^ normal copies [22]. Duplicate reactions were run for each sample on the Mx300P real-time PCR instrument (Stratagene/Agilent), including also positive and negative controls. The PNA-clamped PCR products from samples with an amplification signal for both duplicate reactions were further analyzed by Sanger sequencing as described above, using a *KRAS* exon 2 forward primer designed for sequencing of the PNA-clamped products (Additional file 1: Table S1).

### Statistical analysis

All statistical analyses were conducted in R version 3.5.0 using RStudio version 1.1.423. The R package *MXM* was used to perform a permutation test for Pearson’s correlation with 1 million permutations to account for the small sample size, with the original *p*-value from a student’s *t*-distribution reported as well as the empirical *p*-value from the permutation test. The Mann-Whitney U test was used to assess the difference in the ctDNA level between the cases with a *TP53* mutation detected only in the tumor and the cases with the same *TP53* mutation detected in both the tumor and the juice samples.

## Results

### Detection of *KRAS* mutations in the primary tumor

From our biobank of pancreatic cancer cases [23–25], we identified 21 patients who fulfilled the following criteria: Whipple’s resection performed due to pancreatic head tumor, a verified diagnosis of PDAC, diagnostic FFPE tissue blocks available, and pancreatic juice sample collected during surgery. Clinical characteristics of these cases are presented in Table 1.

We first evaluated *KRAS* mutation status in the primary tumor by Sanger sequencing, a technique which has a limited sensitivity for detection of somatic mutations. Twenty samples (95%) were positive (Table 2). Nineteen of these cases had a mutation in codon 12 and one case had a codon 61 mutation, whereas mutations in codon 13 were not detected. The high frequency of *KRAS* mutations in our patient cohort is consistent with published data on PDAC cases when sensitive detection methods are used [8] and indicated that we had obtained the desired enrichment of tumor cells by manually dissecting regions of interest from the FFPE sections.

**Table 2:**
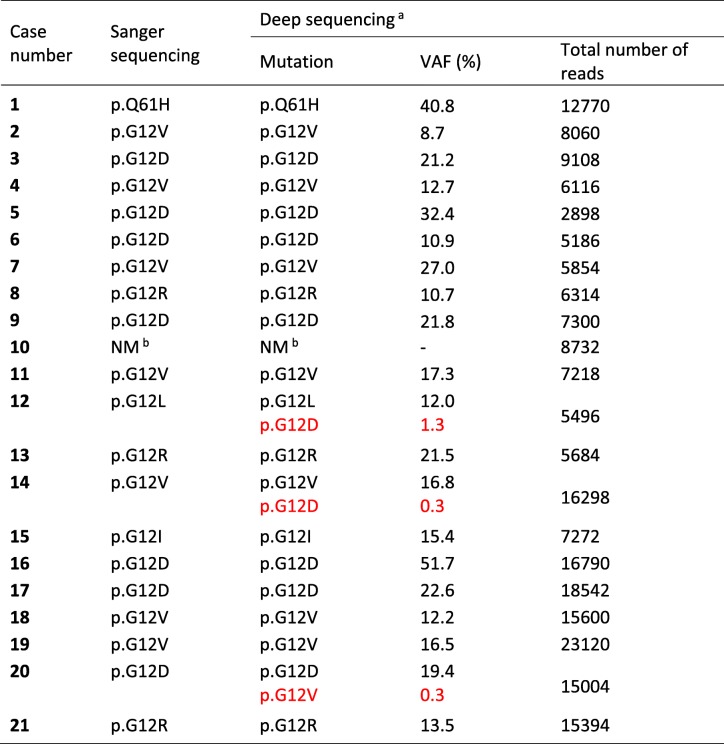
*KRAS* mutation status in the primary tumors as determined by Sanger and deep sequencing

^a^Low-abundance *KRAS* mutations with a variant allele frequency (VAF) < 3% are specified in red color. NM, no mutation

^b^Sample was *KRAS*-negative. Number of reads refers to *KRAS* exon 2. The sample harbored a *BRAF* p.V600E mutation (14.5% VAF, 33228 reads)

*KRAS* mutation status of the primary tumor was then determined by amplicon-based targeted deep sequencing using the Illumina TruSight Tumor 15 panel. For all cases, the mutation status was in concordance with the results from Sanger sequencing (Table 2). VAF varied considerably between cases, from 8.7 to 51.7%. There was limited correlation between VAF and tumor cellularity (*r* = 0.56, *p* = 0.0097, permutation *p* = 0.012). Deep sequencing verified that two cases (#12 and #15) had indels and not biallelic single nucleotide substitutions, as these alternatives were indistinguishable by Sanger sequencing. In 3 cases (#12, #14 and #20), an additional *KRAS* mutation of minor allele frequency (< 3%) was identified by manual examination of the sequencing reads through the IGV tool. Deep sequencing also revealed that the only case with wild-type *KRAS* (#10) harbored the hotspot mutation p.V600E (c.1799 T > A) in *BRAF* exon 15 (Table 2). This mutation was verified by Sanger sequencing.

### Detection of *KRAS* mutations in pancreatic juice

Next, *KRAS* mutations in ctDNA were evaluated by deep sequencing pancreatic juice samples from the 21 cases. When the standard threshold of VAF ≥ 3% was used, 15 cases (71%) were positive (Table 3). However, multiple *KRAS* mutations with VAF below the threshold were observed in many of the juice samples when manually examined by using the IGV tool. When taking these low-frequency variants into account, all samples except one (#17) were positive for one or more *KRAS* mutations (Table 3). To distinguish the low-frequency *KRAS* mutations from technical artefacts, we validated their presence by independently determining the *KRAS* mutation load in pancreatic juice using a PNA clamp real-time PCR assay (see Methods). The PNA clamp assay was positive for *KRAS* exon 2 mutations in the juice samples from the same 20 cases that had been determined positive by deep sequencing (Table 3). The PNA-clamped PCR products were subsequently sequenced and careful comparisons of individually identified *KRAS* exon 2 mutations revealed a high concordance between both detection methods with regard to the observed variants (Table 3). Despite low sensitivity, also direct Sanger sequencing of pancreatic juice DNA visualized the presence of multiple *KRAS* mutations when their frequencies were relatively high (VAF > 5%), such as in cases #7 and #15 (data not shown).

**Table 3:**
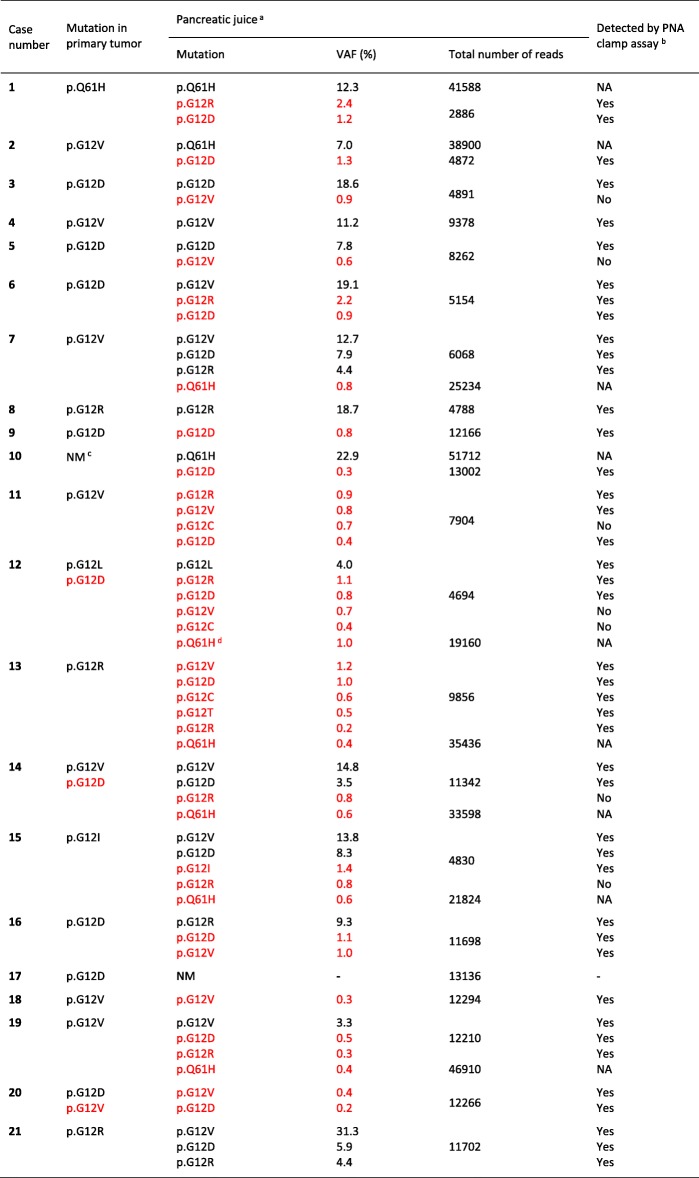
*KRAS* mutation status in the pancreatic juice samples as determined by deep sequencing

^a^Low-abundance *KRAS* mutations with a VAF < 3% are specified in red color. NM, no mutation

^b^NA, not assayed by the PNA clamp method

^c^*KRAS*-negative and *BRAF*-positive tumor. No *BRAF* p.V600E mutation was detected in the corresponding juice sample (23,266 reads at the locus)

^d^Sample had both c.183A > T and c.183A > C mutations, both corresponding to p.Q61H

Thus, our results showed that DNA isolated from pancreatic juice of the PDAC patients frequently harbored *KRAS* mutations, many of which were present at low concentrations or not detected in the primary tumor. Overall, the *KRAS* mutation of the tumor could be observed in the juice DNA in 18 cases (86%) (Table 3). However, the *KRAS* mutation identified in the primary tumor corresponded to the predominating mutation in the juice to a much lesser degree (11 cases, 52%).

### Detection of *TP53* mutations in the primary tumor and in pancreatic juice

Deep sequencing detected a *TP53* mutation in the primary tumor of 16 patients (76%), with VAFs ranging from 6.4 to 49.9% (Table 4). These mutations were all confirmed by Sanger sequencing. The detection rate of *TP53* mutations in our study patients is similar to previously reported data on PDAC [8]. Most of the identified mutations were located in the hotspot exons 5–8 (Additional file 3: Table S3) [26].

**Table 4:**
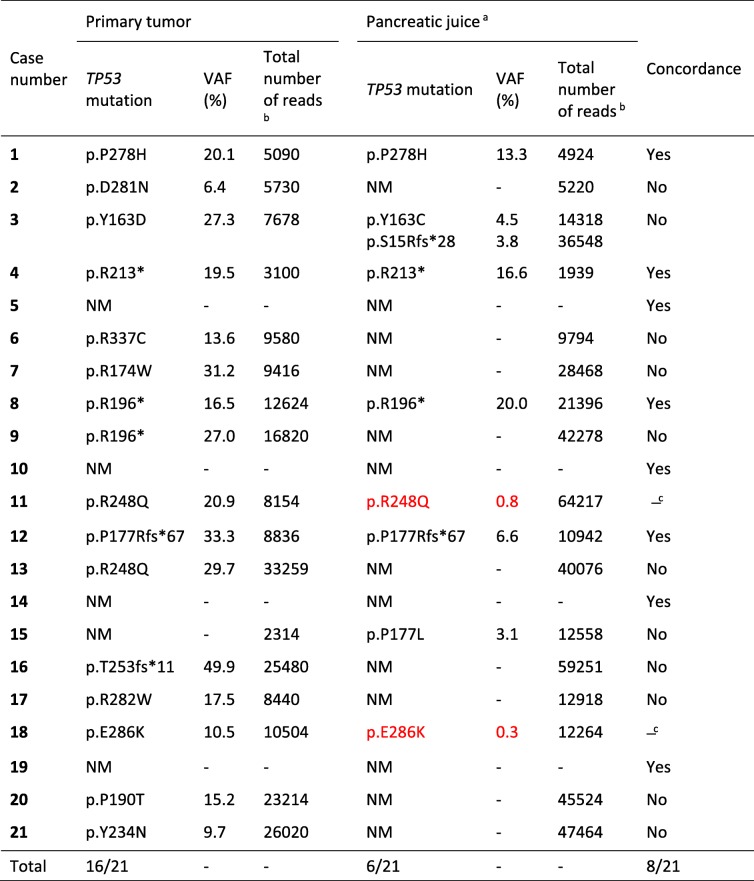
Concordance of *TP53* mutations detected in primary tumor and pancreatic juice by deep sequencing

^a^Low-abundance *TP53* mutations with VAF < 3% are specified in red color. NM, no mutation

^b^Total number of sequencing reads at the *TP53* mutation locus is listed when the mutation was detected in one sample type but not in the other

^c^Concordant only if the low-frequency mutation in the juice sample is considered

Deep sequencing of the DNA samples from pancreatic juice revealed *TP53* mutations only in six patients (29%) when using the 3% VAF threshold (Table 4). In four of these cases, the mutation was identical in the juice sample and primary tumor. We examined if there was an association between ctDNA level and positive *TP53* status in the juice samples. The percentage of the primary tumor-specific *KRAS* mutation in pancreatic juice (Table 3) was then used as a surrogate measure of the amount of ctDNA. We observed that the ctDNA level was significantly lower (*p* = 0.045, Mann-Whitney U test) in those cases where the *TP53* mutation of the primary tumor was not found in the juice (median *KRAS* VAF: 0.8%, range: 0–18.6%) than in those four instances where the *TP53* mutation was detected (cases #1, 4, 8, 12; median *KRAS* VAF: 11.8%, range: 4.0–18.7%).

One patient (#3) harbored more than a single *TP53* mutation in the juice, and neither of the two detected mutations were identical to that seen in the primary tumor. The sixth patient with *TP53*-positive juice sample (#15) had a *TP53*-negative tumor. Altogether, only 8 of 21 cases had exactly the same mutation status (normal sequence or identical mutation) when primary tumor and juice were compared (Table 4). When manually inspecting the deep sequencing data for low-frequency variants, two additional juice samples (#11 and #18) were positive for *TP53* mutations, both containing the same variant as the primary tumor.

### Overall mutation profiles of *KRAS* and *TP53* in the primary tumor and pancreatic juice

**Fig. 1:**
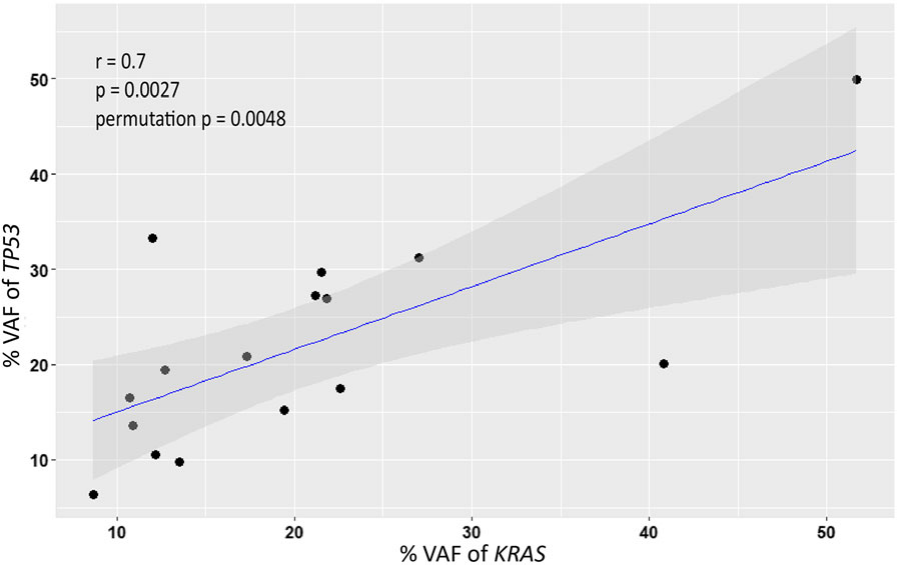
A scatterplot of the variant allele frequency (VAF) of the *KRAS* mutations against that of the *TP53* mutations detected in the primary tumors (*n* = 16). The grey shade represents the 95% confidence interval of the Pearson’s correlation coefficient *r*. The *p*-values are from a student’s *t*-distribution test and from an empirical test with 1 million permutations

Sixteen patients (76%) harbored both *KRAS* and *TP53* mutations in their primary tumor whereas four had only a *KRAS* mutation. The last case was *BRAF*-positive and *TP53*-negative. Pearson’s correlation analysis confirmed a positive correlation between the VAF of both *KRAS* and *TP53* mutations in the primary tumor (*r* = 0.7, *p* = 0.0027, permutation *p* = 0.0048; Fig. 1).

**Fig. 2:**
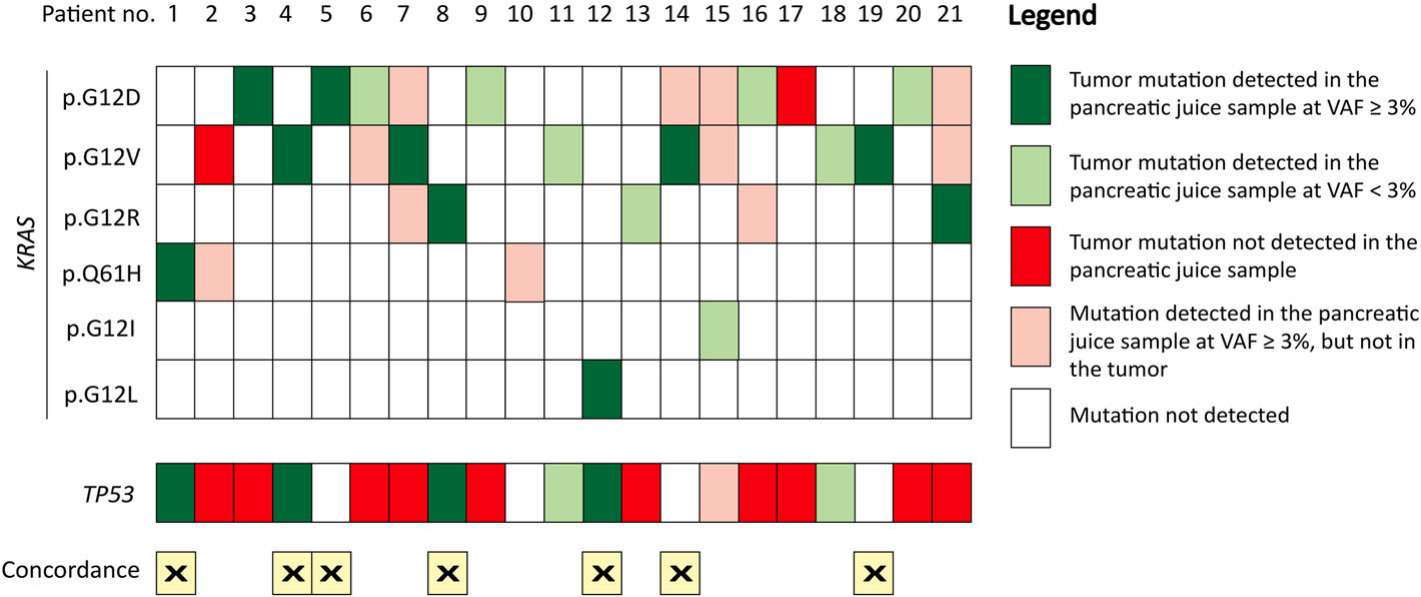
Overall *KRAS* and *TP53* mutation profile in matched primary tumor and pancreatic juice samples from PDAC patients. Color coding indicates relationships between mutations detected in the primary tumor and what was found in the corresponding pancreatic juice. The seven cases in which the mutation status (positive or negative) was concordant in both sample types and for both genes are marked at the bottom of the display. For concordance evaluation, only mutations with a detected variant allele frequency (VAF) ≥ 3% were considered

Results from the juice samples were more complicated with many low-frequency *KRAS* mutations and fewer *TP53* mutations. A summary with regard to *KRAS* and *TP53* mutation status for all cases is presented in Fig. 2. Taken together, parallel analysis of *KRAS* and *TP53* in the primary tumor and pancreatic juice resulted in exactly the same mutation status in 7 of 21 cases (33%) when also concordant *TP53* negativity was considered.

No mutation in the 13 other genes covered by the TruSight Tumor 15 gene panel (Additional file 2: Table S2) was observed in any case, except for the *BRAF* mutation detected in the single primary tumor being *KRAS*-negative. The juice sample from that case did not display any *BRAF* mutation.

## Discussion

Here we have characterized the mutation patterns of *KRAS* and *TP53* in matched pancreatic tumor and juice samples from 21 PDAC patients, using targeted deep sequencing with Sanger sequencing and PNA clamp assay as complementary methods. We identified multiple *KRAS* mutations in the juice DNA from almost all cases (95%). Most of the *KRAS* mutations in pancreatic juice were present at low frequencies (VAF < 3%) and were not seen in the primary tumor.

Previous mutational analyses have shown that *KRAS* mutations are commonly detected in pancreatic juice sampled from patients with pancreatic cancer [9, 13, 27] or from persons undergoing screening because they are considered high-risk subjects [12, 14, 28, 29]. Our observation of multiple *KRAS* mutations in most juice samples is consistent with an earlier report focusing on three hotspot *KRAS* mutations of codon 12 in matched pancreatic juice and tumor specimens [9]. Unlike that study, our method covered the full spectrum of known somatic *KRAS* mutations, occurring in codons 12, 13, 59, 61, 117 and 146 [30], and we demonstrate that the mutation detected in the primary tumor not necessarily was the predominating *KRAS* variant in the patient’s juice sample. Moreover, when the mutation found in the tumor was absent from the juice, other *KRAS* mutations were usually present.

Particularly illustrative in this regard is case #10 with a primary tumor that was *BRAF*-positive and *KRAS*-negative (Table 3). Still, 22.9% of the *KRAS* exon 3 reads from the corresponding juice sample displayed the mutation Q61H, whereas *BRAF* alterations were not detected. An oncogenic *BRAF* mutation is reported to occur in 3% of PDAC cases and is most often mutually exclusive with the presence of a *KRAS* mutation [31]. This is in line with the finding that dysregulation in the RAS-RAF-MAPK signaling pathway is a key driver for PDAC [32]. The absence of the *BRAF* mutation in the juice of case #10 suggests that the fluid contained little DNA arising from the tumor, and that the *KRAS* mutation may have its origin somewhere else, most likely in the tail region of the pancreas drained by the distal duct.

Accordingly, our observation of multiple, mostly low-abundance *KRAS* mutations in pancreatic juice (Table 3), may be explained by the presence of several PanIN precursor lesions in the gland. Low-grade PanIN lesions are frequently present in healthy aged individuals [33] and in PDAC patients [34]. Over 90% of low-grade PanIN-1 lesions have already acquired a *KRAS* mutation [6], but obviously most do not progress to invasive cancer. Nevertheless, these lesions may shed DNA and contribute to the pool of cell-free DNA in the juice. In fact, the presence of more than one *KRAS* mutation in each pancreatic juice sample has been reported from older healthy individuals and patients with pancreatic non-malignant abnormalities such as chronic pancreatitis and cysts [9, 12]. These *KRAS* mutations may dominate over the tumor-specific mutations, as demonstrated in our case series. This strongly suggests that the informative value of detecting *KRAS* mutations in pancreatic juice DNA with the purpose of early pancreatic cancer detection or differential diagnostics is limited. It should be noted, though, that the presence of multiple *KRAS* mutations in a pancreatic juice sample also might reflect clonal heterogeneity of the primary tumor [8].

Detection of *TP53* mutations in combination with *KRAS* in pancreatic juice could improve specificity for PDAC, because somatic alterations in *TP53* arise later during tumorigenesis and is generally present only in high-grade PanIN lesions [6]. Such mutations are in general absent in juice samples from healthy individuals and chronic pancreatitis cases [11]. Thus, with one exception, no juice sample in our series harbored more than a single *TP53* mutation. The striking difference between the *KRAS* and *TP53* mutation distributions lends further support to the assumption that the majority of the multiple *KRAS* mutations found in pancreatic juice DNA originate from low-grade PanIN lesions.

The detection rate of *TP53* mutations (29%) in the juice samples of our study is substantially lower than in other reports studying this biological material from PDAC patients (around 60%) [11, 13, 14]. However, in those publications information of the *TP53* mutation status of the primary tumor was lacking for the majority of cases. Moreover, the pancreatic juice samples stemmed from the duodenal lumen of PDAC patients who had their tumors located in all regions of the pancreas [11, 13, 14]. In contrast, the juice samples of our study were collected from the distal pancreatic duct where the fluid had accumulated due to obstruction imposed by the tumor located in the pancreatic head. This physical obstruction of the proximal pancreatic duct could possibly have favored the relative enrichment of DNA from the distal part of the pancreas rather than from the tumor. Consistently, we observed that the amount of ctDNA was low in those cases where a *TP53* mutation was detected only in the tumor (when using the percentage of the tumor-specific *KRAS* mutation in pancreatic juice as a surrogate measure for ctDNA level). Because of their later occurrence during tumorigenesis, *TP53* mutations generally have a frequency that is lower than or similar to that of *KRAS* during clonal expansion of the cancer [35]. Thus, the scarcity of detected *TP53* mutations in the juice samples could partly be due to a ctDNA level below the detection limit and partly due to the fact that around 30% of PDAC cases lack *TP53* point mutations or small indels [8].

Nevertheless, we found that *TP53* mutations were absent from the majority of the juice samples, even when the tumor was positive. With reference to the mutation profile in the primary tumor, we suggest that analyzing *TP53* mutations in combination with *KRAS* mutations in the juice might represent a more specific although, unfortunately, less sensitive test for PDAC detection.

Our study has several limitations. Firstly, the number of cases was limited and prevented us from investigating the relationship between mutation detection (or concentration) and clinico-pathological variables such as patient survival, cancer stage and tumor differentiation. Secondly, we employed a commercial deep sequencing panel that was constructed to cover 15 genes frequently mutated in various cancers (Additional file 2: Table S2). For PDAC, this panel covers only *KRAS* and *TP53* among the frequently mutated genes in this cancer type. We detected a *BRAF* mutation in one case, but otherwise the remaining 12 genes were negative for all specimens tested. Thirdly, the amplicon-based deep sequencing technology was not optimized to identify low-abundance mutations in the juice. The concentration of ctDNA in pancreatic juice can often be low (VAF < 3%), as shown in our study. This makes it challenging to reliably detect and distinguish low-frequency mutations from PCR artefacts and sequencing errors inherent in amplicon-based assays [36].

For *KRAS* exon 2, we circumvented this issue by using the highly sensitive PNA clamp technology to complement and independently identify low-abundance mutations. The results from the PNA clamp assay supported the deep sequencing results as all samples were positive except one (#17), which was negative with both techniques. Sanger sequencing of the PNA-clamped products also confirmed that multiple *KRAS* mutations were indeed present in DNA from pancreatic juice. However, using complementary assays depending on mutation-specific probes (e.g. PNA clamp assays and droplet digital PCR) is impractical when a larger set of cancer-associated genes are to be screened. Strategies such as digital deep sequencing [13, 14] and molecular barcoding [37] should be implemented in order to better characterize the mutational load in pancreatic juice in future follow-up work. Moreover, sampling of duodenal fluid after secretin stimulation [38] represents a less invasive procedure for obtaining pancreatic juice than sampling directly from the pancreatic duct and would therefore be the method of choice when screening pancreatic cancer high-risk patients. On the other hand, the tumor-specific DNA may then be more diluted, as duodenal juice also contains DNA (including bacterial DNA) and fluid from the duodenal lumen [28].

Finally, we note that in the *KRAS*- and *TP53*-mutated primary tumor cases, the allele frequencies of both mutations tended to be similar (Fig. 1), supporting the view that the two mutations originated from the same tumor clone. We also found that some patients exhibited *KRAS* and/or *TP53* mutations at an allele frequency of around 50% in the tumor (e.g. case #16). As stromal and other non-neoplastic cells will contribute significantly to the isolated DNA, this suggests an amplification event of the oncogenic *KRAS* allele [39] and deletion of the wild-type *TP53* allele [8], respectively. Noteworthy, a subset of pancreatic cancers manifest genomic instability that leads to chromosomal alterations including the *KRAS* and *TP53* loci [40].

## Conclusions

Our results show that pancreatic juice DNA from patients with PDAC is very rich in *KRAS* mutations. Most of these mutations were not present in the primary tumor of the pancreatic head but might reflect somatic mutations within PanIN lesions in other regions of the organ. This supports the notion that detection of only *KRAS* mutations in pancreatic juice samples has limited diagnostic utility in relation to PDAC. The addition of *TP53* mutation detection could result in a more specific test for PDAC, although with reduced sensitivity. Most likely, additional genes (such as the frequently mutated *CDNK2A* and *SMAD4* genes [8]) or biomarkers associated with pancreatic malignancies (such as DNA methylation [41] and telomerase activity [42]) must be included to fully exploit the clinical potential of pancreatic juice samples in early cancer detection.

## Additional files

Additional file 1: **Table S1.** Primers used for PCR amplification and Sanger sequencing of *KRAS*, *TP53* and *BRAF* mutations. (DOCX 21 kb)

Additional file 2: **Table S2.** List of genes and target regions covered by the TruSight Tumor 15 gene panel. (DOCX 19 kb)

Additional file 3: **Table S3.** Interpretation of all *KRAS*, *TP53* and *BRAF* mutations observed in the study. (DOCX 37 kb)

## Abbreviations

*BRAF*: Gene encoding B-Raf proto-oncogene serine/threonine kinase
ctDNA: circulating tumor DNA
FFPE: Formalin-fixed, paraffin-embedded
H&E: Hematoxylin and eosin
IGV: Integrative Genomics Viewer
*KRAS*: Gene encoding Kirsten RAS proto-oncogene GTPase
PanIN: Pancreatic intraepithelial neoplasia
PDAC: Pancreatic ductal adenocarcinoma
PNA: Peptide nucleic acid
*TP53*: Gene encoding tumor suppressor protein p53
VAF: variant allele frequency
