# Supplementary material for: Mutation analysis by deep sequencing of pancreatic juice from patients with pancreatic ductal adenocarcinoma
Source: BMC Cancer. 2019 Jan 5;19:11. doi: 10.1186/s12885-018-5195-7 (PMC6321709; doi:10.1186/s12885-018-5195-7)
Supplement: Supplementary file 1 — Table S1. Primers used for PCR amplification and Sanger sequencing of KRAS, TP53 and BRAF mutations. (DOCX 21 kb) [file 12885_2018_5195_MOESM1_ESM.docx]

| **Table S1**. Primers used for PCR amplification and Sanger sequencing of *KRAS*, *TP53* and *BRAF* mutations. | | | | |
| --- | --- | --- | --- | --- |
| Gene | Exon | Sequence | Amplicon size (bp) | Annealing temperature (°C) |
| *KRAS* | 2 | Forward: 5’-CCTTATGTGTGACATGTTCT | 218 | 57 |
|  |  | Reverse: 5’-ATGGTCCTGCACCAGTAATA |  |  |
|  |  | Seq. Reverse (PNA): 5’-GCCTGCTGAAAATGACTGA | - | 57 |
|  | 3 | Forward: 5’-GGATTCCTACAGGAAGCAAGT | 140 | 60 |
|  |  | Reverse: 5’-TGGCAAATACACAAAGAAAGC |  |  |
|  |  | Seq. Reverse: 5’-TACACAAAGAAAGCCCTCCCCA | - | 55 |
| *TP53* | 5 | Forward: 5’-TTCAACTCTGTCTCCTTCCT | 248 | 60 |
|  |  | Reverse: 5’-CAGCCCTGTCGTCTCTCCAG |  |  |
|  | 6 | Forward: 5’-GCCTCTGATTCCTCACTGAT | 181 | 60 |
|  |  | Reverse: 5’-TTAACCCCTCCTCCCAGAGA |  |  |
|  | 7 | Forward: 5’-ACTGGCCTCATCTTGGGCCT | 174 | 63 |
|  |  | Reverse: 5’-TGTGCAGGGTGGCAAGTGGC |  |  |
|  | 8 | Forward: 5’-TTAAATGGGACAGGTAGGACC | 231 | 55 |
|  |  | Reverse: 5’-TCCACCGCTTCTTGTCCTGC |  |  |
|  | 9 | Forward: 5’-GACAAGAAGCGGTGGAG | 215 | 57 |
|  |  | Reverse: 5’-CGGCATTTTGAGTGTTAGAC |  |  |
|  | 10 | Forward: 5’-CAATTGTAACTTGAACCATC | 260 | 55 |
|  |  | Reverse: 5’-GGATGAGAATGGAATCCTAT |  |  |
| *BRAF* | 15 | Forward: 5’-TGCTTGCTCTGATAGGAAAATG | 228 | 60 |
|  |  | Reverse: 5’-AGCATCTCAGGGCCAAAAAT |  |  |
|  |  | Seq. Forward: 5’-TGTTTTCCTTTACTTACTACACCTCA | - | 59 |
