# Supplementary material for: Mutation analysis by deep sequencing of pancreatic juice from patients with pancreatic ductal adenocarcinoma
Source: BMC Cancer. 2019 Jan 5;19:11. doi: 10.1186/s12885-018-5195-7 (PMC6321709; doi:10.1186/s12885-018-5195-7)
Supplement: Supplementary file 2 — Table S2. List of genes and target regions covered by the TruSight Tumor 15 gene panel. (DOCX 19 kb) [file 12885_2018_5195_MOESM2_ESM.docx]

| **Table S2.** List of genes and target regions covered by the TruSight Tumor 15 gene panel. | |
| --- | --- |
| Gene | Target regions |
| *ATK1* | Exon 3^a^ |
| *BRAF* | Exon 15^a^ |
| *EGFR* | Focal amplification; exons 12^a^, 18, 19, 20, 21 |
| *ERBB2* | Focal amplification; exons 14^a^, 17, 18, 19, 20^a^, 21^a^, 24, 26 |
| *FOXL2* | Exon 1^a^ |
| *GNA11* | Exon 5^a^ |
| *GNAQ* | Exon 5^a^ |
| *KIT* | Exons 8, 9, 10, 11, 13, 14, 17, 18 |
| *KRAS* | Exons 2^a^, 3^a^, 4 |
| *MET* | Focal amplification |
| *NRAS* | Exons 2^a^, 3^a^, 4 |
| *PDGFRA* | Exons 12, 14, 18 |
| *PIK3CA* | Exons 9, 20 |
| *RET* | Exon 16 |
| *TP53* | Full coding sequence |

^a^ Coverage of these exons is partial and targets specific hotspots.

For further details, see https://emea.illumina.com/products/by-type/clinical-research-products/trusight-tumor-15-gene.html
