# Supplementary material for: Mutation analysis by deep sequencing of pancreatic juice from patients with pancreatic ductal adenocarcinoma
Source: BMC Cancer. 2019 Jan 5;19:11. doi: 10.1186/s12885-018-5195-7 (PMC6321709; doi:10.1186/s12885-018-5195-7)
Supplement: Supplementary file 3 — Table S3. Interpretation of all KRAS, TP53 and BRAF mutations observed in the study. (DOCX 37 kb) [file 12885_2018_5195_MOESM3_ESM.docx]

| **Table S3.** Interpretation of all *KRAS*, *TP53* and *BRAF* mutations observed in the study. | | | | | | | | | | | | | | | |
| --- | --- | --- | --- | --- | --- | --- | --- | --- | --- | --- | --- | --- | --- | --- | --- |
| **Gene** | **Reference sequence^a^** | **Nucleotide change** | **Amino acid change** | **Exon** | **Type of mutation** | **Grantham distance^b^** | **Phylop score^c^** | **SIFT^d^** | **PolyPhen^e^** | **COSMIC ID^f^** | **COSMIC - Reported in pancreatic cancer?** | **Reports in IARC TP53 Database^g^** | **dbSNP ID^h^** | **VAF in ExAC^i^ or gnomAD^j^ Database** | **Classification of pathogenicity^k^** |
| ***KRAS*** | ENST00000256078 | c.34G>C  c.34G>T  c.34_35delinsAT  c.34_35delinsAC  c.34_35delinsCT  c.35G>A  c.35G>T  c.183A>C  c.183A>T | p.G12R  p.G12C  p.G12I  p.G12T  p.G12L  p.G12D  p.G12V  p.Q61H  p.Q61H | 2  2  2  2  2  2  2  3  3 | Missense  Missense  Indel  Indel  Indel  Missense  Missense  Missense  Missense | 125  159  135  59  138  94  109  24  24 | 5.86  5.86  5.86  5.86  5.86  5.86  5.86  3.34  3.34 | Deleterious (0.02)  Deleterious (0.01)  Deleterious (0)  Deleterious (0)  Deleterious (0)  Deleterious (0)  Deleterious (0.01)  Deleterious (0.01)  Deleterious (0.01) | Possibly damaging (0.562)  Probably damaging (0.993)  Probably damaging (0.985)  Probably damaging (0.960)  Probably damaging (0.960)  Benign (0.361)  Probably damaging (0.978)  Benign (0.046)  Benign (0.046) | COSM518  COSM516  COSM34144  COSM1583122  COSM514  COSM521  COSM520  COSM554  COSM555 | Yes  Yes  Yes  No  Yes  Yes  Yes  Yes  Yes | -  -  -  -  -  -  -  -  - | rs121913530  rs121913530  -  -  -  rs121913529  rs121913529  rs17851045  rs17851045 | -  < 1x10^-4^  ^-^  ^-^  ^-^  < 1x10^-4^  -  -  - | 5  5  5  4/5  5  5  5  5  5 |
| ***TP53*** | ENST00000269305 | c.45_48del | p.S15Rfs*28 | 2 | Frameshift | - | - | - | - | COSM5622809 | No | - | - | - | 4 |
|  |  | c.487T>G | p.Y163D | 5 | Missense | 160 | 3.16 | Deleterious (0) | Probably damaging (1) | COSM44216 | No | 5 | rs786203436 | - | 4 |
|  |  | c.488A>G | p.Y163C | 5 | Missense | 194 | 2.22 | Deleterious (0) | Probably damaging (1) | COSM10808 | Yes | 166 | rs148924904 | - | 4 |
|  |  | c.520A>T | p.R174W | 5 | Missense | 101 | 0.96 | Deleterious (0) | Probably damaging (0.997) | COSM44782 | Yes | 16 | - | - | 3 |
|  |  | c.530C>T | p.P177L | 5 | Missense | 98 | 4.04 | Deleterious (0) | Probably damaging (0.992) | COSM44097 | No | 34 | rs751477326 | < 1x10^-4^ | 3 |
|  |  | c.530_539del | p.P177Rfs*67 | 5 | Frameshift | - | - | - | - | - | - | - | - | - | 4 |
|  |  | c.568C>A | p.P190T | 6 | Missense | 38 | 3.98 | Deleterious (0) | Possibly damaging (0.711) | COSM44438 | No | 6 | - | - | 3 |
|  |  | c.586C>T | p.R196* | 6 | Nonsense | - | 2.05 | - | - | COSM10705 | Yes | 240 | rs397516435 | < 1x10^-4^ | 5 |
|  |  | c.637C>T | p.R213* | 6 | Nonsense | - | 1.88 | - | - | COSM10654 | Yes | 321 | rs397516436 | - | 5 |
|  |  | c.700T>A | p.Y234N | 7 | Missense | 143 | 1.23 | Deleterious (0) | Probably damaging (0.995) | COSM43956 | No | 16 | - | ^-^ | 4 |
|  |  | c.743G>A | p.R248Q | 7 | Missense | 43 | 3.65 | Deleterious (0.01) | Probably damaging (0.995) | COSM10662 | Yes | 937 | rs11540652 | < 1x10^-4^ | 5 |
|  |  | c.757_758insA | p.T253fs*11 | 7 | Frameshift | - | - | - | - | COSM45313 | No | - | - | - | 4 |
|  |  | c.833C>A | p.P278H | 8 | Missense | 77 | 3.81 | Deleterious (0) | Probably damaging (1) | COSM43755 | Yes | 15 | - | - | 4 |
|  |  | c.841G>A | p.D281N | 8 | Missense | 23 | 5.39 | Deleterious (0) | Probably damaging (0.989) | COSM43596 | Yes | 35 | rs764146326 | < 1x10^-4^ | 4 |
|  |  | c.844C>T | p.R282W | 8 | Missense | 101 | 1.87 | Deleterious (0) | Probably damaging (1) | COSM10704 | Yes | 581 | rs28934574 | < 1x10^-4^ | 3 |
|  |  | c.856G>A | p.E286K | 8 | Missense | 56 | 3.55 | Deleterious (0) | Probably damaging (0.982) | COSM10726 | No | 96 | rs786201059 | - | 4 |
|  |  | c.1009C>T | p.R337C | 10 | Missense | 180 | 1.07 | Deleterious (0) | Benign (0.344) | COSM11071 | Yes | 20 | rs587782529 | - | 4 |
| ***BRAF*** | ENST00000288602 | c.1799T>A | p.V600E | 15 | Missense | 121 | 5.10 | Deleterious (0) | Possibly damaging (0.943) | COSM476 | Yes | - | rs113488022 | < 1x10^-4^ | 5 |

^a^ Ensembl Genome Database (<https://www.ensembl.org>).

^b^ Physiochemical distance for prediction of the effect of substitutions between amino acids [1].

^c^ Measure of evolutionary conservation at individual alignment sites [2].

^d^ Prediction score for the effect of substitution mutation on protein function (<http://sift.bii.a-star.edu.sg/>) [3].

^e^ Prediction score for the effect of substitution mutation on protein structure and function (<http://genetics.bwh.harvard.edu/pph2/>) [4].

^f^ COSMIC Cancer Database (<http://cancer.sanger.ac.uk/cosmic>).

^g^ International Agency for Research on Cancer (IARC) TP53 Database (<http://p53.iarc.fr>).

^h^ Single Nucleotide Polymorphism database (<https://www.ncbi.nlm.nih.gov/projects/SNP/>).

^i^ Exome Aggregation Consortium (<http://exac.broadinstitute.org/>).

^j^ Genome Aggregation Database (<http://gnomad.broadinstitute.org/>).

^k^ Classification according to the American College of Medical Genetics and Genomics (ACMG) guidelines: class 5 (pathogenic), class 4 (likely pathogenic) and class 3 (variants of uncertain significance) [5]

**Supplementary References**

1. Grantham, R. *Amino acid difference formula to help explain protein evolution.* Science 1974. 185: 862-4.

2. Pollard, KS et al. *Detection of nonneutral substitution rates on mammalian phylogenies.* Genome Res. 2010. 20: 110-21.

3. Kumar P et al. *Predicting the effects of coding non-synonymous variants on protein function using the SIFT algorithm*. Nat. Protoc. 2009;4:1073-81.

4. Adzhubei, I et al. *Predicting functional effect of human missense mutations using PolyPhen-2.* Curr. Protoc. Hum. Genet. 2013. Chapter 7: Unit7.20.

5. Richards, S et al. *Standards and guidelines for the interpretation of sequence variants: a joint consensus recommendation of the American College of Medical Genetics and Genomics and the Association for Molecular Pathology.* Genet. Med. 2015. 17: 405-24.
